# Renal osteodystrophy and clinical outcomes: a prospective cohort study

**DOI:** 10.1590/2175-8239-JBN-2023-0119en

**Published:** 2023-10-30

**Authors:** Cinthia Esbrile Moraes Carbonara, Joaquim Barreto, Noemi Angelica Vieira Roza, KélciaRosana da Silva Quadros, Luciene Machado dos Reis, Aluízio Barbosa de Carvalho, Andrei C. Sposito, Vanda Jorgetti, Rodrigo Bueno de Oliveira

**Affiliations:** 1 Universidade Estadual de Campinas, Faculdade de Ciências Médicas, Divisão de Nefrologia, Campinas, SP, Brazil.; 2 Universidade Estadual de Campinas, Laboratório de Biologia Vascular e Aterosclerose, Campinas, SP, Brazil.; 3 Universidade de São Paulo, Faculdade de Medicina, Laboratório de Fisiopatologia Renal, São Paulo, SP, Brazil.; 4 Universidade Federal de São Paulo, São Paulo, SP, Brazil.; 5 Universidade Estadual de Campinas, Faculdade de Ciências Médicas, Laboratório para Estudo do Distúrbio Mineral e Ósseo em Nefrologia (LEMON), Campinas, SP, Brazil.

**Keywords:** Chronic Kidney Disease-Mineral and Bone Disorder, Renal Osteodystrophy, Renal Insufficiency, Chronic, Clinical Outcomes, Distúrbio Mineral e Ósseo na Doença Renal Crônica, Osteodistrofia Renal, Insuficiência, Renal Crônica, Desfechos Clínicos

## Abstract

**Introduction:**

Renal osteodystrophy (ROD) refers to a group of bone morphological patterns that derive from distinct pathophysiological mechanisms. Whether the ROD subtypes influence long-term outcomes is unknown. Our objective was to explore the relationship between ROD and clinical outcomes.

**Methods:**

This study is a subanalysis of the Brazilian Registry of Bone Biopsies (REBRABO). Samples from individual patients were classified as having osteitis fibrosa (OF), mixed uremic osteodystrophy (MUO), adynamic bone disease (ABD), osteomalacia (OM), normal/minor alterations, and according to turnover/mineralization/volume (TMV) system. Patients were followed for 3.4 yrs. Clinical outcomes were: bone fractures, hospitalization, major adverse cardiovascular events (MACE), and death.

**Results:**

We enrolled 275 participants, of which 248 (90%) were on dialysis. At follow-up, 28 bone fractures, 97 hospitalizations, 44 MACE, and 70 deaths were recorded. ROD subtypes were not related to outcomes.

**Conclusion:**

The incidence of clinical outcomes did not differ between the types of ROD.

## Resumo

**Introdução:**

Osteodistrofia renal (OR) refere-se a um grupo de padrões morfológicos ósseos que decorrem de mecanismos fisiopatológicos distintos. É desconhecido se os subtipos de OR influenciam desfechos em longo prazo. Nosso objetivo foi explorar as relações entre OR e desfechos.

**Métodos:**

Este estudo é uma subanálise do Registro Brasileiro de Biópsias Ósseas (REBRABO). As amostras de cada paciente foram classificadas em osteíte fibrosa (OF), osteodistrofia urêmica mista (MUO), doença óssea adinâmica (ABD), osteomalácia (OM), alterações normais/menores, e pelo sistema Remodelação / Mineralização / Volume (RMV). Os pacientes foram acompanhados por 3,4 anos. Os eventos clínicos foram: fraturas ósseas, hospitalizações, eventos cardiovasculares adversos maiores (MACE), e óbito.

**Resultados:**

Analisamos 275 indivíduos, 248 (90%) deles estavam em diálise. No acompanhamento, 28 fraturas ósseas, 97 hospitalizações, 44 MACE e 70 óbitos foram registrados. Os subtipos de OR não foram relacionados aos desfechos clínicos.

**Conclusão:**

A incidência de desfechos clínicos não diferiu entre os tipos de OR.

## Introduction

Renal osteodystrophy (ROD) refers to a group of bone morphological changes due to chronic kidney disease (CKD) that are classically classified as osteitis fibrosa, mixed uremic osteodystrophy, adynamic bone disease, and osteomalacia, and/or by the turnover / mineralization / volume (TMV) system^1^.

Each of these patterns is not only histologically different, but also derive from distinct pathophysiological mechanisms^1,2^. For example, differences in bone turnover, which is a classifying feature of ROD variety, may influence vascular calcification and hence the risk of cardiovascular disease, the leading cause of death among CKD subjects^3^.

The hypothesis that the ROD variety may influence the incidence of outcomes was previously tested by our group, with a relatively short mean follow-up^4^. Nevertheless, whether ROD subtypes are evenly related to long-term outcomes is unknown.

To address this question, we hereby present the results of a subanalysis of the *Brazilian Registry of Bone Biopsy* (REBRABO)^5^, in which patients with ROD were followed by 3.4 years and hard outcomes were assessed. Of note, to the best of our knowledge, this is the first study to assess the influence of ROD subtypes on long-term morbimortality.

## Methods

This study was conducted as a subanalysis of the REBRABO data^5^, and is related in part to previously published data^4,6,7,8^. The detailed methodology has been described elsewhere^4–8^. Briefly, the REBRABO is a prospective cohort of patients with ROD. This research was carried out from August 15 to December 21. Bone samples from patients with CKD were classified, using the conventional classification (recognition of histological patterns), as having osteitis fibrosa (OF), mixed uremic osteodystrophy (MUO), adynamic bone disease (ABD), osteomalacia (OM), normal/minor alterations, and according to the Turnover / Mineralization / Volume (TMV) system. Patients were followed for an average of 1242 (693-1508) days, or 3.4 yrs. Clinical events reported were bone fractures, hospitalization, major adverse cardiovascular events (MACE; unstable angina, nonfatal acute myocardial infarction, elective or emergency coronary revascularization, transient ischemic attack, stroke, and cardiovascular death), and death. Cox regression analysis was used to detect covariates and factors associated with outcomes. The study was approved by the ethics committee (number 4131141.6.0000.5404), and patients provided written informed consent.

## Results

We enrolled 275 patients in this subanalysis, of which 248 (90%) were on dialysis. OF was diagnosed in 113 (41%) patients, ABD in 79 (29%), MUO in 59 (21%), OM in 12 (4%), and normal/minor alterations in 12 (4%). Table 1 shows the characteristics of the patients at baseline according to the main outcome recorded duringfollow-up. Of note, patients who were lost to follow-up (N = 111) had similar characteristics to the sample of this subanalysis (Table S1).

**Table 1:** Characteristics of the patients at baseline according to the main outcome recorded during follow-up

	All	Survivors	Deceased	p
	(N = 275)	(N = 205)	(N = 70)	
Age (years)	52 (42–60)	50 (41– 58)	56 (50–64)	**0.0001**
BMI (kg/m^2^)	24.1 (22–27)	24.7 (22–27)	24 (22–27)	0.92
Male (N, %)	143 (52)	104 (51)	39 (56)	0.47
Caucasian (N, %)	118 (43)	86 (42)	32 (46)	0.58
DM (N, %)	39 (14)	25 (12)	14 (20)	0.10
Previous PTx (N, %)	46 (17)	40 (19)	6 (9)	**0.03**
Previous CVD (N, %)	27 (10)	14 (7)	13 (19)	**0.004**
CKD etiology				0.05
AH (N, %)	78 (28)	59 (29)	19 (27)	
CGN (N, %)	65 (24)	51 (25)	14 (20)	
DM (N, %)	37 (13)	21 (10)	16 (23)	
Dialysis vintage (months)	84 (36–146)	84 (36–144)	77 (38–171)	0.83
Hemodialysis (N, %)	221 (80)	165 (90)	56 (86)	0.37
Hemoglobin (g/dL)	11.5 (10.3–13)	11.6 (10.3–13.2)	11.2 (10.3–12.1)	0.06
Total calcium (mg/dL)	9.3 (8.6–9.8)	9.3 (8.6–9.9)	9.2 (8.8–9.8)	0.93
Phosphate (mg/dL)	5 (3.9–6.5)	4.9 (3.9–6.3)	5.1 (3.7–6.5)	0.91
Parathormone (pg/mL)	234 (65–733)	238 (58–752)	217 (82–544)	0.97
Alkaline phosphatase (IU/L)	120 (79–217)	118 (76–211)	132 (83–239)	0.27
25-vitamin D (ng/mL)	29.6 (20.5–38)	31 (22–38)	26.3 (19.2–35.8)	0.39

BMI, body mass index; DM, Diabetes *Mellitus*; PTx, parathyroidectomy; CVD, cardiovascular disease; AH, arterial hypertension; CGN, chronic glomerulonephritis.

During the follow-up, 28 bone fractures, 97 hospitalizations, 44 MACE, and 70 deaths were recorded, corresponding to an annual incidence of 4.4%, 14.6%, 6.8%, and 7.5%, respectively. The proportion of ROD types was similarly distributed across the outcomes (Table 2).

**Table 2:** Proportion of renal osteodystrophy and incidence of clinical outcomes

	Bone fracture			Hospitalization			MACE			Death		
Renal osteodystrophy diagnosis	No	Yes	p	No	Yes	p	No	Yes	p	No	Yes	p
Osteitis fibrosa (N; %)	64 (41)	14 (50)	0.36	43 (43)	38 (39)	0.54	62 (43)	17 (39)	0.62	87 (42)	26 (37)	0.43
Mixed uremic osteodystrophy (N; %)	26 (17)	7 (25)	0.28	17 (17)	20 (21)	0.54	25 (17)	9 (20)	0.63	42 (20)	17 (24)	0.50
Adynamic bone disease (N; %)	51 (32)	7 (25)	0.43	28 (28)	34 (35)	0.30	46 (32)	14 (32)	0.99	57 (28)	22 (31)	0.56
Osteomalacia (N; %)	6 (4)	0 (0)	NA	4 (4)	2 (2)	0.68	4 (3)	2 (4)	0.62	9 (4)	3 (4)	1.0
Normal/Minor alterations (N; %)	10 (6)	0 (0)	NA	7 (7)	3 (3)	0.33	8 (5)	2 (4)	1.0	10 (5)	2 (3)	0.73

MACE, major adverse cardiovascular events; NA, non-applicable.

Patients who presented bone fractures had similar characteristics to those without fractures. Patients who were hospitalized were older [52 (47–60) *vs*. 48 (40–58) yrs.; p = 0.03] and presented lower serum hemoglobin levels [11.5 (10–13) *vs*. 12.2 (10.7–13.7; p = 0.02]. Low serum hemoglobin levels were independently associated with hospitalization [OR: 0.903 (CI: 0.823–0.991)]. Patients who presented MACE had lower serum hemoglobin levels [11.1 (9.6–12.6) *vs*. 12 (10.8–13.5; p = 0.026], increased prevalence of DM [11 (25%) *vs*. 15 (10%); p = 0.01], and previous CVD [8 (18%) *vs*. 8 (5%); p = 0.008]. DM was an independent predictor ofMACE [OR: 3.287 (CI: 1.541–7.011)].

Compared to survivors, patients who died were older [56 (50–64) *vs*. 50 (41–58) yrs.; p < 0.0001], had increased prevalence of CVD [13 (19%) *vs*. 14 (7%); p = 0.004], fewer had phosphate levels in the reference range [17 (24%) *vs*. 80 (39%), p = 0.026] and fewer had parathyroidectomy [6 (9%) *vs*. 40 (19%); p = 0.03]. Age, previous CVD, and proportion of patients with serum phosphate levels outside the reference range were independent predictors of death [OR: 1.046 (CI: 1.024–1.069), p = 0.0001; OR: 1.856 (CI: 1.009–3.413), p = 0.04; OR: 1.942 (CI: 1.116–3.379), p = 0.019; respectively].

**Figure 1:**
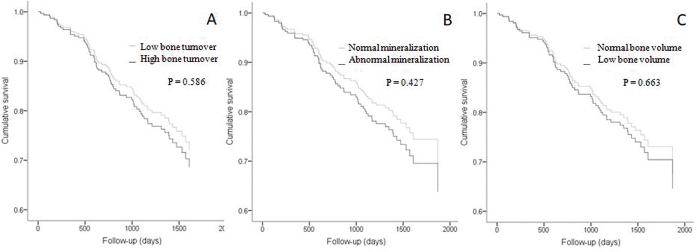
Effects of bone turnover, mineralization, and volume on death outcome. Cox regression analysis survival curves for death outcome. The variables tested in the models were age, previous cardiovascular disease, previous parathyroidectomy, proportion of patients outside of the reference range for serum phosphate levels, and bone turnover in (A), bone mineralization in (B), or bone volume in (C). Overall p = 0.0001.

Different models of Cox regression analysis with OF, MUO, ABD, OM, or bone TMV parameters did not reveal ROD as an independent predictor of hospitalization, MACE, or death (Figure 1).

## Discussion

We observed an annual incidence of bone fractures, hospitalization, MACE, and death of 4.4%, 14.6%, 6.8%, and 7.5%, respectively. The incidence of these outcomes did not differ according to ROD types.

Compared to our previous report,^4^ the follow-up time was doubled, and the number of patients increased from 115 to 275. However, we did not detect any effect of the different patterns of ROD on these outcomes.

Of note, the annual mortality rate in this cohort (7.5%) was lower than that reported by national surveys, which registered an average estimated annual crude mortality rate indialysis patients of about 19% in the last 5 years^9^. These data suggest that bone histology of patients with ROD can impact clinical decisions and may be associated with lower death rates.

This study had some limitations. It was an essentially descriptive study, and the sample was not randomly selected. The impact of treatments based on ROD diagnosis on outcomes was not measured, and extrapolation of these findings to other populations is not possible. Nephrologists in charge of each patient indicated and performed the bone biopsy attheir discretion or based on a research protocol. They were also the ones who entered the baseline data into the REBRABO system. Outcomes were assessedby telephone calls with the dialysis unit staff and patients. These facts can introduce unavoidable bias. The strength of our study was the prospective nature, with data from a cohort of patients with ROD, which is unusual. Our study is the first to access the effects of ROD on hard outcomes, with a rather long follow-up.

## Conclusions

In this prospective cohort, the incidence of adjudicated outcomes did not differ between the patterns of ROD.

## Supplementary Material

The following online material is available for this article:

Table s1 - General and biochemical data according to follow-up.

## References

[B1] Moe S, Drüeke T, Cunningham J, Goodman W, Martin K, Olgaard K (2006). Kidney Disease: Improving Global Outcomes (KDIGO). Definition,
evaluation, and classification of renal osteodystrophy: a position statement
from Kidney Disease: Improving Global Outcomes (KDIGO).. Kidney Int..

[B2] Drüeke TB, Massy ZA (2016). Changing bone patterns with progression of chronic kidney
disease.. Kidney Int..

[B3] Bover J, Ureña P, Brandenburg V, Goldsmith D, Ruiz C, DaSilva I (2014). Adynamic bone disease: from bone to vessels in chronic kidney
disease.. Semin Nephrol..

[B4] Carbonara CEM, Reis LM, Quadros KRS, Roza NAV, Sano R, Carvalho AB (2020). Renal osteodystrophy and clinical outcomes: data from the
Brazilian Registry of Bone Biopsies – REBRABO.. J Bras Nefrol..

[B5] Oliveira RB, Barreto FC, Custódio MR, Gueiros JE, Neves CL, Karohl C (2014). Brazilian Registry of Bone Biopsy (REBRABO): design, data,
elements, and methodology.. J Bras Nefrol..

[B6] Carbonara CEM, Roza NAV, Quadros KRS, França RA, Esteves ABA, Pavan CR (2023). Effect of aluminum accumulation on bone and cardiovascular risk
in the current era.. PLoS One..

[B7] Carbonara CEM, Roza NAV, Reis LMD, Carvalho AB, Jorgetti V, Oliveira RB (2023). Overview of renal osteodystrophy in Brazil: a cross-sectional
study.. Braz J Nephrol..

[B8] Carbonara C, Quadros K, Roza N, Barreto J, Reis L, Carvalho A (2023). Renal osteodystrophy and clinical outcomes: results from The
Brazilian Registry of Bone Biopsy - Abstract presented in the 60th ERA
Congress.. Nephrol Dial Transplant..

[B9] Nerbass FB, Lima HN, Thomé FS, Vieira No OM, Lugon JR, Sesso R (2022). Brazilian Dialysis Survey 2020.. J Bras Nefrol..

