# Supplementary material for: Renal osteodystrophy and clinical outcomes: a prospective cohort study
Source: J Bras Nefrol. 2023 Oct 30;46(2):e20230119. doi: 10.1590/2175-8239-JBN-2023-0119en (PMC11210535; doi:10.1590/2175-8239-JBN-2023-0119en)
Supplement: Supplementary file 1 [file 2175-8239-jbn-2023-0119-s1.pdf]

Braz. J. Nephrol. (São Paulo)
https://doi.org/10.1590/2175-8239-JBN-2023-0119en

Supplementary Material of "Renal osteodystrophy and clinical outcomes: a prospective cohort study"

TABLE S1 General and biochemical data according to follow-up status.

	With follow-up (N = 275)	Lost follow-up (N = 111)	p
Age (years)	52 (42–60)	50 (39 – 60)	0.38
BMI (kg/m^2)	24.1 (22–27)	24 (21-27)	0.44
Male (N, %)	143 (52)	55 (49)	0.66
Caucasian (N, %)	118 (43)	42 (38)	0.36
DM (N, %)	39 (14)	18 (16)	0.61
Previous CVD (N, %)	27 (10)	9 (8)	0.60
Previous PTx (N, %)	46 (17)	27 (24)	0.08
CKD etiology			0.31
AH (N, %)	78 (28)	27 (24)	
CGN (N, %)	65 (24)	29 (26)	
DM (N, %)	37 (13)	9 (8)	
Dialysis vintage (months)	84 (36–146)	96 (51-168)	0.17
Hemodialysis (N, %)	221 (80)	94 (96)	0.06
Hemoglobin (g/dL)	11.5 (10.3–13)	11.5 (10-13)	0.77
Total calcium (mg/dL)	9.3 (8.6–9.8)	9.3 (8.6-10.1)	0.70
Phosphate (mg/dL)	5 (3.9–6.5)	4.8 (3.6-6)	0.15
Parathormone (pg/mL)	234 (65–733)	220 (55-930)	0.49
Alkaline phosphatase (IU/L)	120 (79–217)	129 (82-257)	0.20
25-vitamin D (ng/mL)	29.6 (20.5–38)	28.2 (22.2-36.6)	0.89

BMI, body mass index; DM, Diabetes *Mellitus*; PTx, parathyroidectomy; CVD, cardiovascular disease; AH, arterial hypertension; CGN, chronic glomerulonephritis.
